# X-ray dark-field computed tomography for monitoring of tissue freezing

**DOI:** 10.1038/s41598-024-56201-3

**Published:** 2024-03-07

**Authors:** Dominik John, Wolfgang Gottwald, Daniel Berthe, Sami Wirtensohn, Julia Hickler, Lisa Heck, Julia Herzen

**Affiliations:** 1 Research Group Biomedical Imaging Physics, Department of Physics, TUM School of Natural Sciences, Technical University of Munich, 85748 Garching, Germany; 2 Chair of Biomedical Physics, Department of Physics, TUM School of Natural Sciences, Technical University of Munich, 85748 Garching, Germany; 3 Munich Institute of Biomedical Engineering, Technical University of Munich, 85748 Garching, Germany; 4 Institute of Materials Physics, Helmholtz-Zentrum hereon, 21502 Geesthacht, Germany

**Keywords:** Preclinical research, Biomedical engineering, X-rays, Preclinical research, Biomedical engineering, X-rays

## Abstract

Accurately monitoring the extent of freezing in biological tissue is an important requirement for cryoablation, a minimally invasive cancer treatment that induces cell death by freezing tissue with a cryoprobe. During the procedure, monitoring is required to avoid unnecessary harm to the surrounding healthy tissue and to ensure the tumor is properly encapsulated. One commonly used monitoring method is attenuation-based computed tomography (CT), which visualizes the ice ball by utilizing its hypoattenuating properties compared to unfrozen tissue. However, the contrast between frozen and unfrozen tissue remains low. In a proof-of-principle experiment, we show that the contrast between frozen and unfrozen parts of a porcine phantom mimicking breast tissue can be greatly enhanced by acquiring X-ray dark-field images that capture the increasing small-angle scattering caused by the ice crystals formed during the procedure. Our results show that, compared to X-ray attenuation, the frozen region is detected significantly better in dark-field radiographs and CT scans of the phantom. These findings demonstrate that X-ray dark-field imaging could be a potential candidate for improved monitoring of cryoablation procedures.

## Introduction

Cryoablation, a local ablative technique used in interventional oncology mainly for malignancies of the liver, kidney, prostate, lung and breast, has recently received increased interest^1^. In this procedure, cell death is induced by freezing tissue using a cryoprobe inserted percutaneously near the tumor. Compared to conventional surgical resection, which can lead to chronic pain, infection, hematoma and seroma formation, cryoablation has several advantages. Due to the natural analgesic effect of cold, it is usually painless and can be performed with only local anesthesia. Scarring is reduced due to the small needle hole and the patient can be discharged the same day^2^. However, the extent of the treated regions must be monitored during the procedure to minimize damage to healthy tissue while freezing the tumor region with a safety margin of 8–10 mm^3,4^. Unnecessarily large frozen regions have been shown to lead to fat necrosis, infection, and skin injury^5,6^.

For the treatment of breast cancer, the procedure has been repeatedly shown to be an effective option for tumors smaller than 15 mm in size^7–9^. The most popular methods for monitoring breast cryoablation procedures are ultrasound (US) and attenuation-based computed tomography (CT)^2,10^, although magnetic resonance imaging (MRI)-guided procedures have also been successfully performed^11^. While US-guided monitoring allows visualization of the anterior surface of the frozen region, the posterior margin cannot be resolved due to acoustic shadowing^8,12^. For this reason, CT or MRI guidance is recommended in addition to US to provide a safe distance from critical structures in the thorax^11^. Real-time US imaging has been reported as the most operator-dependent technique, as it requires considerable practice and knowledge of the artifacts that occur^10^.

Because soft tissue contrast is inherently low for X-ray attenuation imaging, imaging techniques that use the phase shift and/or scattering of the X-ray wavefront passing through the tissue as an additional contrast mechanism are the subject of ongoing research. Such approaches include propagation-based imaging^13–15^, which has been successfully applied for breast radiographies and CTs at synchrotron sources, as well as grating-based imaging (GBI)^16–19^ and coded apertures^20,21^. The latter two approaches have been demonstrated to be compatible with conventional X-ray sources and detectors. GBI has the additional advantage of simultaneously providing a dark-field, phase-contrast, and attenuation signal in a single scan by modulating the X-ray beam using up to three gratings with absorbing or phase-shifting properties^22^. In this context, dark-field is defined as a signal associated with the local visibility reduction of the interferometer due to local small-angle scattering caused by spatially random unresolved microstructure.

In recent years, it has been shown that the additional information provided by phase-contrast and dark-field modalities is beneficial for the detection of lesions and microcalcifications in the breast^23–27^. However, the dark-field signal may not only be useful for diagnosis, but also for the monitoring of freezing processes: We propose that frozen tissue, causing strong X-ray scattering due to the presence of ice crystals, should be well distinguishable from unfrozen tissue, which causes comparatively little scattering. This seems plausible as the effect has been previously used for distinguishing between frozen and unfrozen fruit^28^. In the currently used attenuation-based CT-guidance, only the slight change in the density of the frozen tissue, which leads to a hypoattenuating region, is used as a contrast mechanism^29^.

The potential of X-ray dark-field monitoring for the freezing of tissue models has so far not been investigated in either radiography or computed tomography. In this work, we show that the extent of the frozen region in a porcine phantom mimicking breast tissue can be monitored more accurately with a grating interferometer at a laboratory X-ray source than with attenuation-based imaging alone. For this purpose, the phantom is actively frozen using a pipe connected to a liquid nitrogen reservoir. The imaging results are first demonstrated in radiographs and then transferred to a CT setup.

## Results

### Radiography

**Figure 1:**
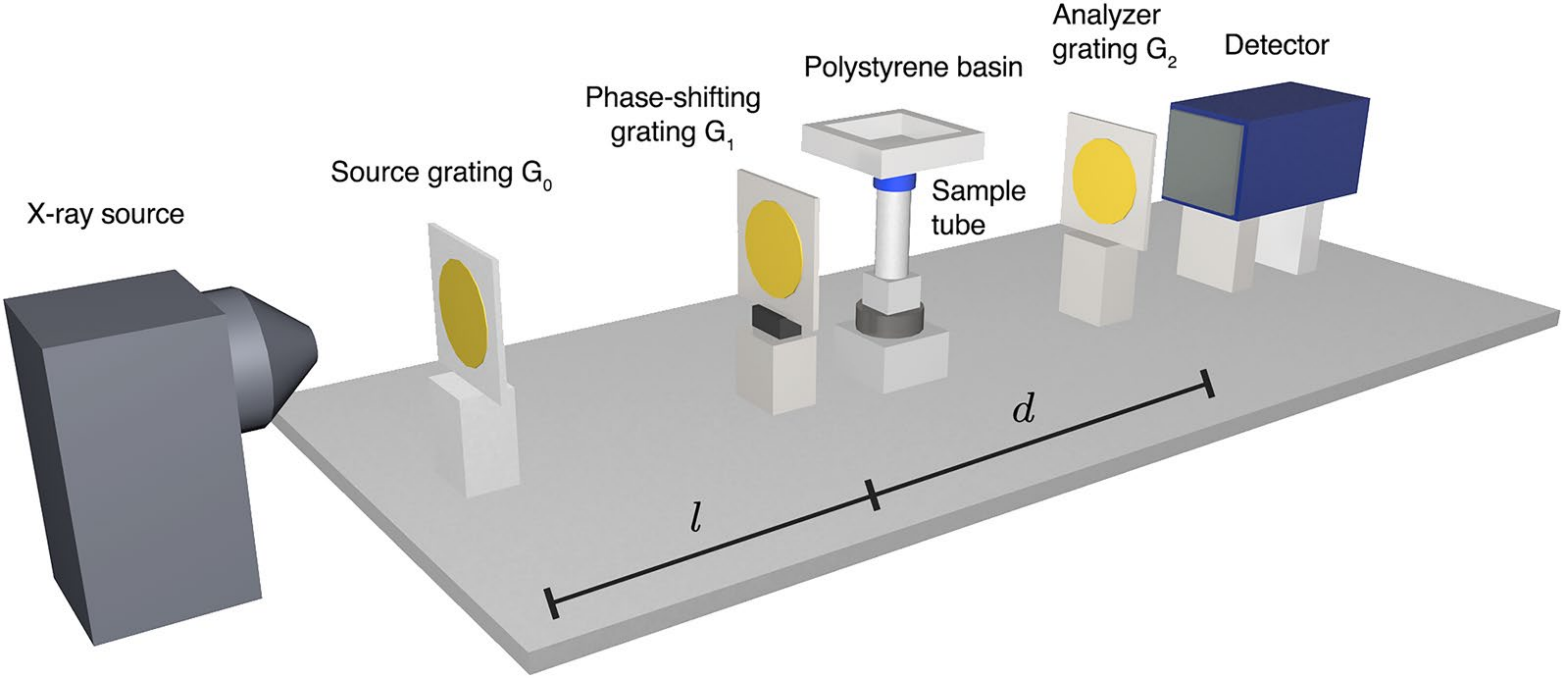
Sketch of the experimental setup used for the measurements. The X-ray source is followed by the source grating G_0_ and the phase-shifting grating G_1_. Next, the beam passes through the sample tube, which is either cooled in the center by liquid nitrogen flowing from a basin through a thin pipe (CT experiments) or using a copper rod connected to a liquid nitrogen reservoir on one end (radiography experiments). The X-rays then reach the analyzer grating G_2_ and the beam is absorbed by a detector. The distances *l* and *d* mark the distances G_0_–G_1_ and G_1_–G_2_ respectively.

**Figure 2:**
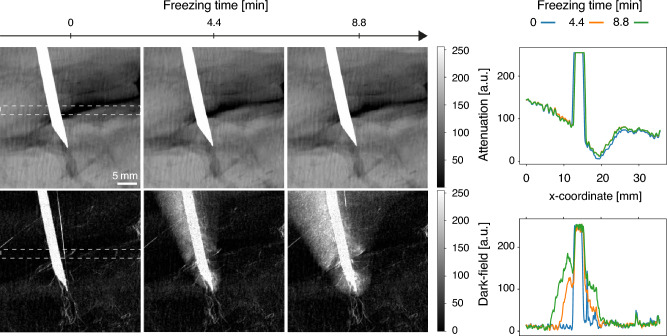
Series of radiographs (left) and line plots (right) of a pork neck slab frozen using a copper rod connected to a liquid nitrogen reservoir on one end. While the extent of the frozen region is clearly visible in the dark-field images, it is not visually detectable in the attenuation images. The line plots show the signal change over time in the dotted white rectangular region, averaged in the vertical direction.

First, a piece of pork neck is frozen using a copper rod cooled by liquid nitrogen, the setup being illustrated in Fig. 1. The process is monitored using grating-based radiographs acquired using 11 grating-phase steps, each with an exposure time of 6 s, resulting in a total acquisition duration of 66 s per radiograph. Three of the resulting pairs of attenuation and dark-field images at different times are shown along with their accompanying line plots in Fig. 2. It may be observed that the attenuation images show no change over time while the dark-field images clearly show the expansion of the frozen region around the needle. This is most likely caused by the formation of ice crystals in the tissue, leading to increased small-angle scattering and therefore increased dark-field signal. The signal shows a decreasing intensity from the rod outwards, corresponding to the density of ice crystals. The line plot of the attenuation shows no significant change. A small lateral shift of the intensity values corresponding to the copper rod is most likely caused by a slight contraction of the sample during the freezing. In the dark-field line plot, the expansion of the frozen region is easy to detect as well. The average dose per radiograph is 4.4 mGy—each radiograph simultaneously providing information for both a dark-field and attenuation image.

### Computed tomography

**Figure 3:**
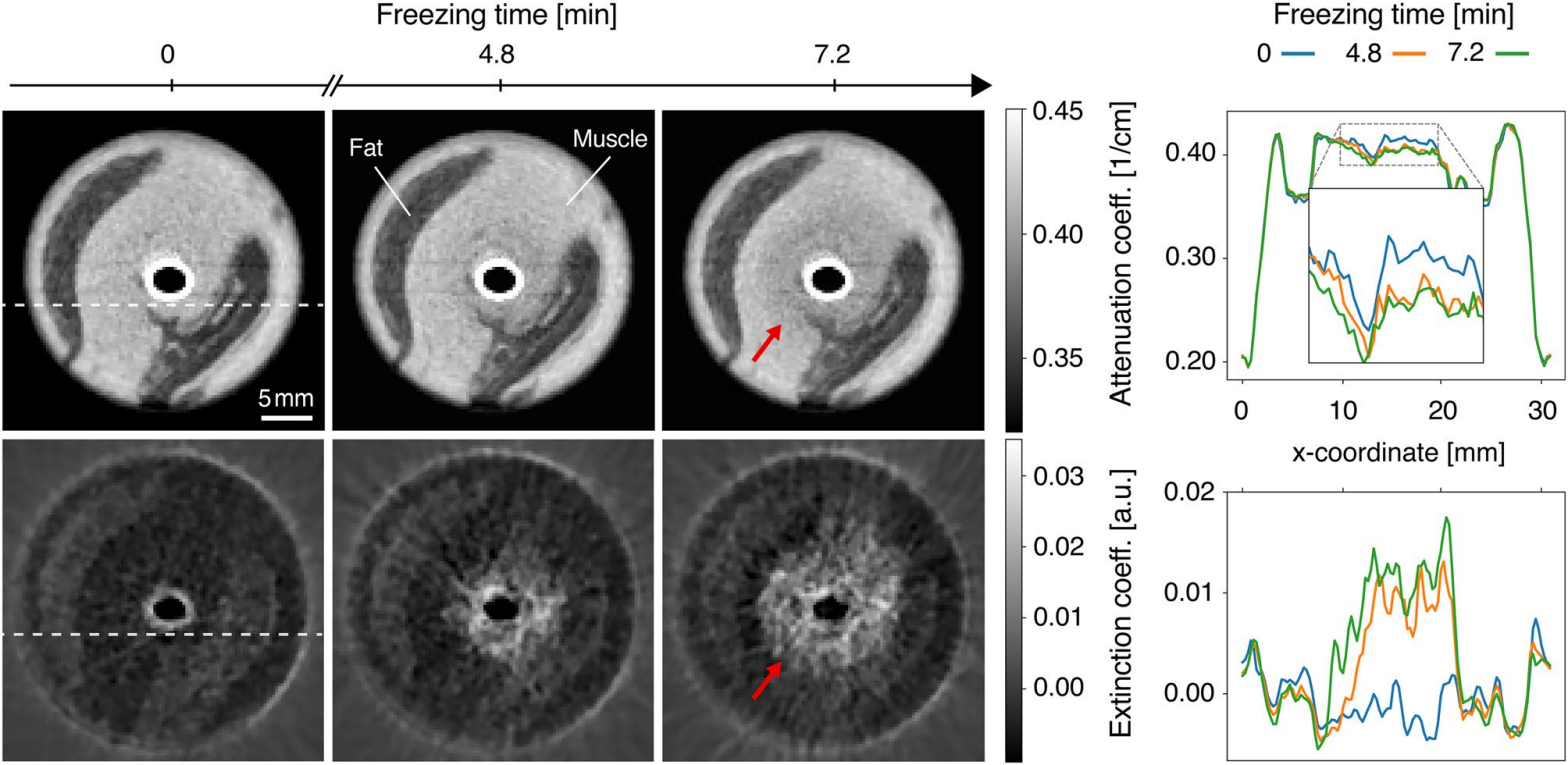
Series of axial slices (left) and line plots (right) near the top of the sample in dark-field and attenuation images at different time points. A radially symmetric increase in signal strength is observed in the dark-field while the same region appears slightly darker in the attenuation images. The red arrows mark the boundary of the frozen region, which is easier to see in dark-field. The acquisition time for each multimodal CT scan is 2.4 min. The line plots show the intensity along the dotted white line, averaged over 10 axial slices extending beyond the depicted image plane.

**Figure 4:**
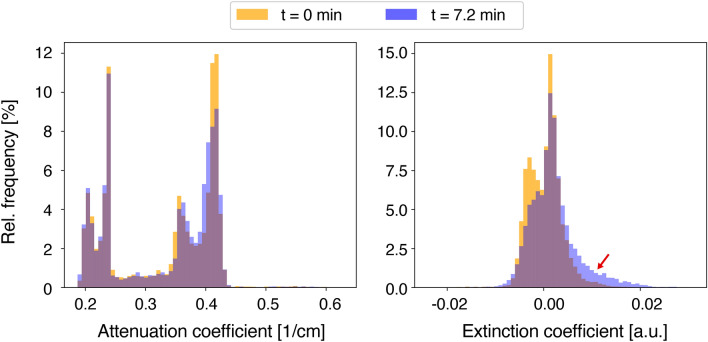
Overlayed histograms for the attenuation image slice (left) and extinction coefficient image slice (right) from Fig. 3. The image slices correspond to the leftmost (orange) and rightmost (blue) column of Fig. 3 respectively. While the attenuation signal shows a slight shift of values towards the left, the extinction values are shifted by a larger magnitude towards the right (red arrow).

Next, the process is transferred from radiography to three-dimensional imaging. For this purpose, ten consecutive grating-based CT scans are performed while a piece of pork belly is being frozen. Compared to the radiography, a significantly lower exposure time of 0.12 s per phase step is chosen to enable sufficient time resolution. For each CT scan, the sample completes a 360^∘^ rotation within 2.4 min; the mean glandular dose is estimated at 22 mGy. Freezing is induced by the flow of liquid nitrogen through a thin tube pierced through the center of the sample. Figure 3 shows an axial slice of the sample over time in the attenuation and dark-field modalities together with plots of the intensity values along the dotted white line. In the dark-field, a radially symmetric increase in signal intensity over time is observed near the wall of the central cooling tube. A change in signal intensity is also observed in the attenuation images, although it is more difficult to detect. Here, a hypoattenuating region extends radially outward from the cooling tube. As indicated by the red arrows, the boundary of this region is less sharply defined than the boundary seen in the corresponding dark-field images. Histograms of the image slices in the first and third column are provided in Fig. 4 to show that the dark-field signal experiences a larger change in contrast compared to the attenuation signal, regardless of the specific windowing chosen in Fig. 3.

The dark circle in the center of all images corresponds to the inside of the polytetrafluoroethylene (PTFE) cooling tube, which is mostly filled with evaporating liquid nitrogen. Its walls produce a visible signal in both the attenuation and dark-field images. In terms of artifacts, the dark-field images show streaks caused by the strong scattering signal present in the frozen region. They are approximately radially symmetric and brighten the background outside the frozen region over time. The attenuation images display streaking artifacts in the form of two faint streaks running horizontally from the longer sides of the elliptically compressed rod.

**Figure 5:**
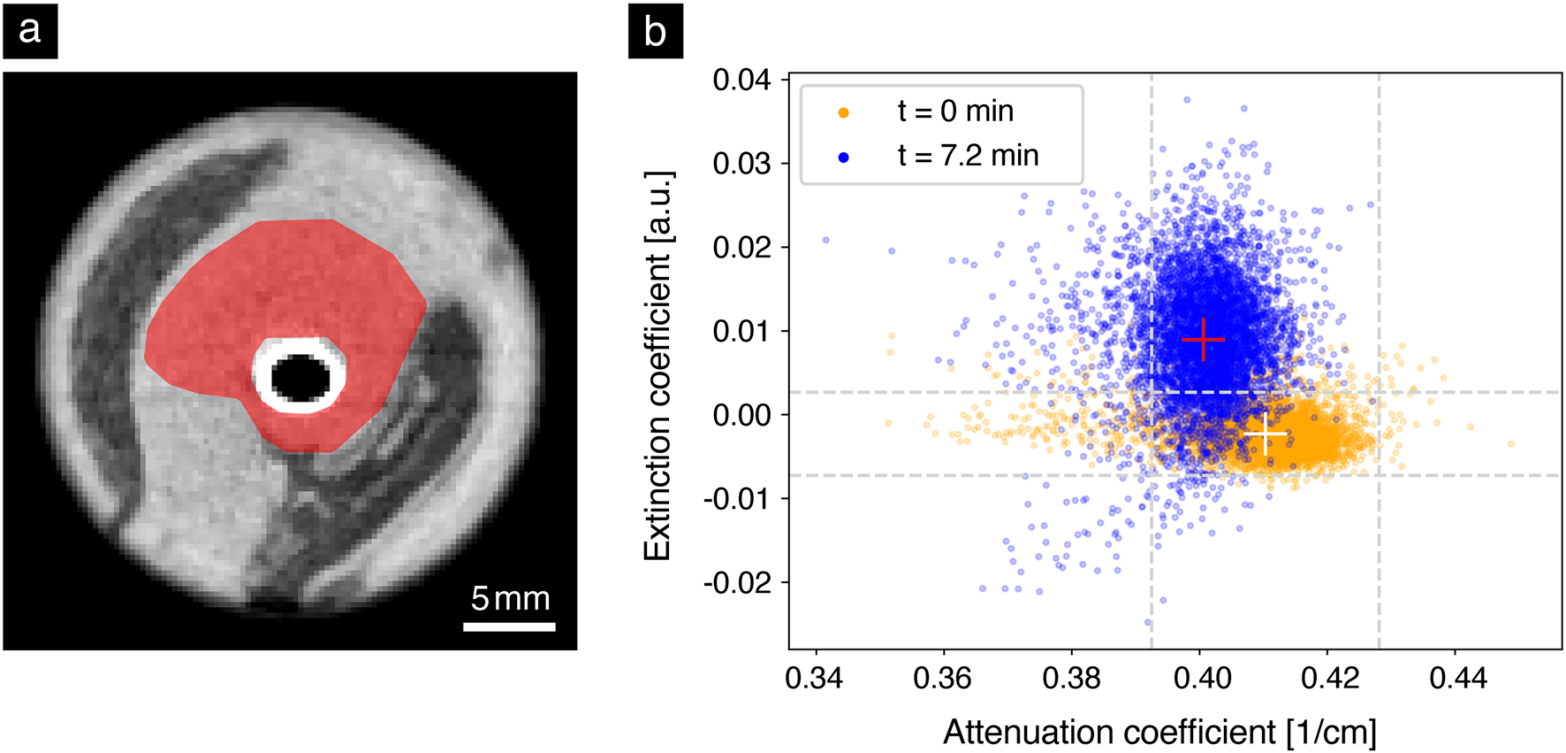
Analysis of the change in attenuation and extinction coefficients over time in muscle tissue. (**a**) Region of interest (red) used for the analysis depicted in (**b**). The cooling tube and a neighboring fat region (dark) remain unselected to create a homogeneous region. It extends over 10 axial slices beyond the depicted image plane. (**b**) Plot of the attenuation and extinction coefficient values of all voxels in the region of interest defined in (a) at time $t = {0}\,{\hbox {min}}$ (orange) and after 7.2 min (blue). The dashed gray lines indicate the positions of the mean signal in each channel $\pm 2\sigma$ at $t = {0}\,{\hbox {min}}$, $\sigma$ being the standard deviation. During the freezing process, the original center of mass (white cross) moves to a new position (red cross) by a vertical distance greater than $2\sigma$. This means that the average extinction coefficient experiences a significant increase. In comparison, the average attenuation coefficient decreases insignificantly and remains below $2\sigma$.

To examine how easily freezing is detected in the two imaging modalities, a region of interest as shown in Fig. 5a is selected and analyzed. The maximum radius corresponds to the largest distance at which signs of freezing were observed during the time window of the experiment. The region excludes the elliptical region bounded by the edge of the cooling tube, which is not relevant for the freezing analysis. To create a region with mostly homogeneous attenuation coefficients, the fat region (dark) is also excluded. This allows tracking changes in one specific tissue without creating an overlap with results from other tissues. Figure 5b shows a scatter plot of the attenuation and dark-field signal of each voxel in the region of interest at time $t = {0}\,{\hbox {min}}$ and at $t = {7.2}\, {\hbox {min}}$. The mean extinction coefficient value at the beginning of the experiment is $-\,0.002 \pm 0.002$ a.u. During the freezing process, higher extinction coefficient values are reached and the distribution widens in the vertical direction. Overall, the center of mass of the distribution is significantly ($> 2 \sigma )$ shifted upward, where $\sigma$ corresponds to the standard deviation. A shift of the center of mass towards lower attenuation coefficients is also observed. However, this shift is within $2\sigma$ of the original distribution. These differences in the magnitude of the shift underline numerically that the freezing process is easier to detect in the dark-field.

**Figure 6:**
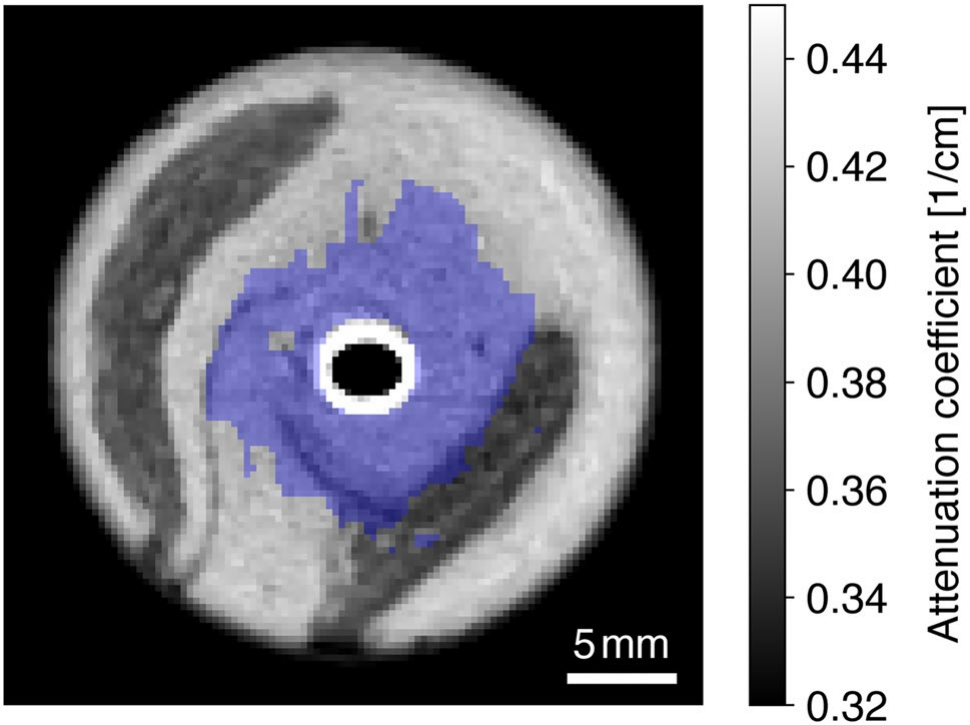
Fusion image of dark-field and attenuation for an example image slice. The binary map (blue) marks voxels as frozen if they experienced an increase in the extinction coefficient of more than $2\sigma$ compared to the mean of the extinction coefficients of all voxels at the beginning (excluding the tube border and the cooling pipe). A morphological closing with a (3,3) kernel was performed to reduce the impact of noise on the visual appearance. Dark-field information from inside the cooling pipe and the edge of the sample tube was discarded using circular masks, as it is not caused by freezing, but by the scattering properties of the materials.

This result is then used as a method to classify a given voxel as frozen depending on its dark-field value. For this purpose, a threshold is defined by adding $2\sigma$ to the mean of the extinction coefficients of all voxels at time $t = {0}\,{\hbox {min}}$, excluding the sample tube edge and the cooling pipe. In Fig. 6, a binary map (blue) of all dark-field voxels that satisfy this condition at $t = {7.2}\,{\hbox {min}}$ is superimposed on the corresponding attenuation image. The result is a single image that combines anatomical (attenuation) and functional (dark-field) information.

## Discussion

In this proof-of-concept work, we have examined the suitability of X-ray attenuation and dark-field signals for tracking an active freezing process in a porcine phantom mimicking breast tissue. We have come to the conclusion that dark-field imaging allows for detecting the frozen region with higher significance than attenuation alone. Compared to attenuation, which shows little change during the freezing process, the dark-field signal has a contrast mechanism better suited to the monitoring task: At room temperature, muscle and fat tissue do not cause significant small-angle scattering and therefore provide little dark-field signal, which changes when ice crystals form during freezing. The method was exemplarily tested on tissue mimicking the composition of the human breast, but may offer similar advantages for the monitoring of freezing in other soft tissue areas (e.g. liver, kidney, prostate). It is likely less suitable for tissue types that already create strong small-angle scattering in the unfrozen state, as is the case for lung tissue^30^. A limitation of our study is the studied sample thickness, which required at most 3 cm depth of X-ray penetration. For a successful translation to the monitoring of real cryoablation procedures, larger sample sizes will need to be studied for their impact on image quality and measurement times.

Moreover, we showed that the dark-field information can be superimposed on the simultaneously acquired attenuation image to provide an image that combines anatomical and functional information, similar to the color overlays used in Doppler ultrasonography^31^. In this experiment, a threshold of $2\sigma$ was used to classify a voxel as frozen. A possible improvement to the visualization would be to display images at multiple thresholds depending on the application: A lower threshold image may show more frozen voxels than are actually present, to avoid accidentally freezing adjacent healthy tissue. A higher threshold image provides even more confidence that the voxels containing the tumor are sufficiently frozen.

In the radiography experiment, the freezing occurred more rapidly on the left side of the needle than on the right (see Fig. 2). This effect is most likely explained by the higher proportion of fat tissue on the right side (visible as a darker region in the attenuation channel), which has a thermal conductivity half that of muscle tissue^32^. This is consistent with the results shown for the CT freezing experiment in Fig. 6, where the frozen region extends further out in muscle tissue compared to fat tissue, as would be expected for the lower thermal conductivity of fat.

It is to be noted that the dark-field CT measurements yield slightly negative extinction coefficients for some pixels. This can be attributed in part to the high amount of noise in the measured projections: If the visibility of a certain pixel in the reference image is decreased due to noise, the visibility may increase by chance in the corresponding sample pixel. This then leads to a positive $V_\text {sample}/V_\text {ref}$ quotient, interpreted as a negative extinction coefficient. Another contributing factor is the fact that extinction coefficients obtained via least-squares fitting of the stepping curve are biased: They are underestimated in measurements with low photon counts^33^, as is the case in our experiment.

A disadvantage of dark-field CT-guided imaging and CT-guided imaging in general compared to ultrasound and MRI is the ionizing radiation to which patients are exposed. In our experiment, each CT scan yielded a dose of 22 mGy, but multiple scans are needed to monitor the procedure over time. As our experiment only constitutes a proof of principle, a number of steps still need to be taken to decrease the dose. Firstly, the exposure time could be reduced by increasing the effective pixel size via additional binning. Since the medically relevant distances for safety margins to organs are in the range of 8–10 mm^3,4^, a doubling of the effective pixel size from 290 to 580 $\upmu {\hbox {m}}$ may still be acceptable. Additionally, the total dose can be lowered by increasing the time in between CT scans, sacrificing temporal resolution, or by decreasing the exposure time further while implementing self-supervised deep-learning denoising to differentiate the frozen border even in very high-noise settings^34^. A limitation of the dose estimate used for this experiment is that the estimate is calculated using a dosimetry method developed for the human breast, although the sample is only similar in composition.

Recently, the feasibility of a human-scale dark-field CT scanner has been demonstrated by adding phase-shifting gratings to the source and absorption gratings near the detector of an existing commercial CT^35^. Measurement times of few seconds have been achieved in this approach by using the intrinsic vibrations of a commercial CT scanner to sample the phase-stepping curve. This demonstrates that the time resolution of 2.4 min of our CT experiment, which was limited due to the applied measurement protocol, can in principle be drastically improved. Factors helping with the adoption of the proposed cryoablation monitoring method would be the equipping of existing, conventional CT scanners with gratings or the development of a dark-field CT arm. Attenuation-based breast CTs, which have been shown to be feasible for breast cancer screening^36^, could also be equipped with gratings to allow intraoperative use for monitoring.

## Methods

### Setup and geometry

The experimental setup is shown in Fig. 1 and uses a rotating anode (MicroMax HF007, *Rigaku Corporation*, Tokyo, Japan) with a molybdenum target as an X-ray source. The variables G_i_ refer to the *i*-th grating in the beam direction. The gratings for both experiments are detailed in Table 1. The geometries of the setups used for radiograph and CT measurements differ slightly due to an upgrade performed to the setup to enable fast CT imaging. The distances and resulting geometric magnifications are provided in Table 2.

For the radiography experiment, the source is operated with a voltage of 33 kVp and a current of 24 mA. A scintillation-based energy integrating detector (Dexela 1512, *PerkinElmer, Inc.*, Waltham, United States) with a pixel pitch of 74.8 $\upmu {\hbox {m}}$ and 1944 $\times$ 1536 pixels is used. The effective pixel size of the setup in this configuration is 53.2 $\upmu {\hbox {m}}$.

For the CT experiment, the source is set to a voltage of 50 kVp and a current of 24 mA. The projections are acquired using a prototype photon-counting detector (Santis 0808 GaAs HR, *DECTRIS AG*, Baden, Switzerland) with a module thickness of 500 $\upmu {\hbox {m}}$ and 1030 $\times$ 1024 pixels with a pitch of 75 $\upmu {\hbox {m}}$. Due to the geometric magnification of the setup, the effective pixel size is 58 $\upmu {\hbox {m}}$. The grating is moved using a P-620.1CD piezoelectric actuator controlled by an E-709.1C1L digital controller (both *Physik Instrumente (PI) GmbH & Co. KG*, Karlsruhe, Germany).

**Table 1:** Grating types used for the radiographs and CT measurements respectively.

Experiment	Role	Type	Period [${{\upmu }}$m]	Substrate material	Substrate height [μm]
Radiograph	G_0_	Absorbing	10	Au	35
Radiograph	G_1_	Phase-shifting	6.5	Ni	9.5
Radiograph	G_2_	Absorbing	4.8	Au	25
CT	G_0_	Absorbing	10	Au	150
CT	G_1_	Phase-shifting	6.5	Ni	9.5
CT	G_2_	Absorbing	4.8	Au	89

The G_1_ grating is identical for both experiments, while G_0_ and G_2_ were replaced by gratings with higher substrate heights for the CT experiments as part of the setup upgrade.

**Table 2:** Setup geometries used for the radiographs and CT measurements respectively.

Experiment	Source–$\text {G}_0$ [mm]	$\text {G}_0$–$\text {G}_1$ [mm]	$\text {G}_1$–Sample [mm]	$\text {G}_1$–$\text {G}_2$ [mm]	$\text {G}_2$–Det. [mm]	Magnification
Radiograph	109	964	85	464	98	1.41
CT	85	1075	160	520	30	1.30

The distances $\text {G}_0$–$\text {G}_1$ and $\text {G}_1$–$\text {G}_2$ are also commonly referred to in the literature as *l* and *d* respectively. The sample position refers to the middle of the sample holders.

### Radiography measurement protocol

Instead of the sample tube shown in Fig. 1, a piece of pork neck is held in place by two polycarbonate plates. To begin the experiment, a copper rod is inserted into the sample and cooled using a polystyrene bath filled with liquid nitrogen. Grating-based radiographs are then taken at regular intervals: Each radiograph consists of 11 phase steps of 6 s exposure time each. The exposure time is chosen to result in a mean glandular dose estimate on the order of the mean glandular dose in two-view digital mammography^37^. The frame time $T_\text {F}$ is approximately equal to the exposure time $T_\text {E} = {6} \,{\hbox {s}}$, because the idle time $T_\text {I}$ of the detector is chosen to be negligibly small. This differs from the CT measurement protocol described in detail in the next subsection. A total of 20 radiographs are taken over a period of 22 min. The time marks in the Fig. 2 refer to the starting time of the acquisition of a new image.

### CT measurement protocol

**Figure 7:**
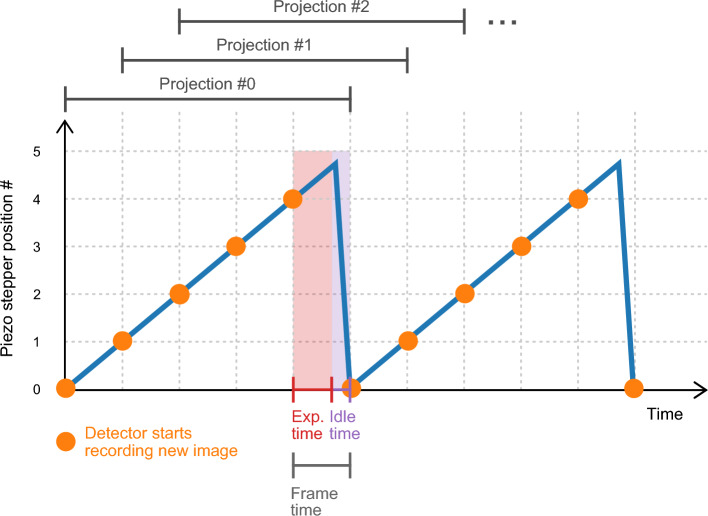
Illustration of the measurement protocol used for the CT scans. The graph shows the movement of the piezoelectric actuator over time while the sample is being rotated. The orange dot indicates the start of a new detector frame of duration $T_\text {F}$, which consists of an exposure time $T_\text {E}$ and an idle time $T_\text {I}$. Photons are only counted during the exposure time. Per 360^∘^ rotation of the sample, 130 ramps are produced by the actuator. The stepping curve provided by 5 consecutive frames is later used in the processing step to calculate one projection. By shifting the starting point by one frame and using the consecutive frames again, the next projection is calculated in a sliding-window fashion.

In order to capture the changes in the sample over time in CT scans, a measurement protocol similar to Zanette et al.^38^ is used. The piezoelectric actuator that controls the position of the $\text {G}_1$ grating is moved continuously in a ramp-like pattern over time while synchronized to the detector. Per 360^∘^ rotation of the sample, the detector records a total of 650 frames with a frame time of $T_\text {F}$ = 0.22 s each, resulting in a full sample rotation duration of 2.4 min. Four additional frames from the next round are used in the processing step to obtain the last projection. Each frame consists of $T_\text {E}$ = 0.12 s exposure time and $T_\text {I}$ = 0.10 s idle time. The idle time, during which no photons are recorded, is present to allow the actuator to return to its original position after the fourth exposure without imaging the resulting motion artifacts. Each frame contains the same idle time to ensure equal spacing of the recorded images. The value of the idle time is chosen to be as small as possible considering the maximum motion speed of the actuator. The exposure time is chosen as a trade-off between the available image statistics and the need for fast rotations to resolve tissue changes over time. Two actuator ramps and the corresponding detector actions are shown in Fig. 7. Due to the periodicity of the phase grating, stepper position #5 produces the same pattern as position #0.

Before beginning the recording, liquid nitrogen is filled into the polystyrene basin at the top of the Falcon^TM^ tube. It then flows through a hollow polytetrafluoroethylene (PTFE) tube with an outer diameter of 3 mm and an inner diameter of 2 mm, which is placed inside the sample and sealed at the bottom with silicone to prevent direct contact of the liquid nitrogen with the tissue. This tube material was chosen to avoid streaking artifacts that would occur with a metal rod. Once the reservoir is filled, the measurement is initiated by recording 10 full ramp movements of the actuator without the sample in the beam to be used for the flat field correction later on. The sample is then moved into the beam path, the rotation is started and the measurement is performed as described earlier. In our experiment, the sample is rotated continuously for 10 rounds of 360^∘^.

### Processing

After the flat-field correction^39^, a weighted least-squares fit of sine curves to the measurements in each pixel is performed to extract the attenuation, dark-field, and phase-contrast signals. The dark-field signal *D* is calculated as

1 $$D =  - \log \frac{{V_{{{\text{sample}}}} }}{{V_{{{\text{ref}}}} }},  $$

where $V_\text {sample}$ is the visibility in a given pixel with the sample in the beam and $V_\text {ref}$ is the visibility without the sample. The attenuation signal is obtained analogously by substituting the visibilities for the measured photon counts. For the CT measurements, the phase retrieval is performed in a sliding-window fashion: The recorded detector frames numbered #0 to #4 are used to obtain projection #0, then the frames #1 to #5 yield the next processed projection #1, as shown in Fig. 7. The phase retrieval is additionally performed in a patch-wise manner, with the patches consisting of the center pixel and its 4 direct neighbors, to mitigate the effects of noise on the phase retrieval^40^. After this step, the data is binned by a factor of 5, resulting in a new effective pixel size of 290 $\upmu {\hbox {m}}$. A ring artifact correction is applied to all signals in the projection space. The dark-field signal is corrected as described in a subsequent subsection to mitigate the effects of beam hardening. A bilateral filter as described by Allner et al. is applied to both signals to reduce the amount of noise present due to the low dose while preserving most of the edges^41^. For the attenuation image, the phase-contrast data is provided to the bilateral filter algorithm as additional edge information.

Because the sensor of the energy-integrating detector used for the radiographs is affected by electronic noise, a dark-current correction is applied in addition to the flat-field correction. The radiographs are neither binned nor filtered, i.e., the resolution corresponds to the effective pixel size.

### Samples

**Figure 8:**
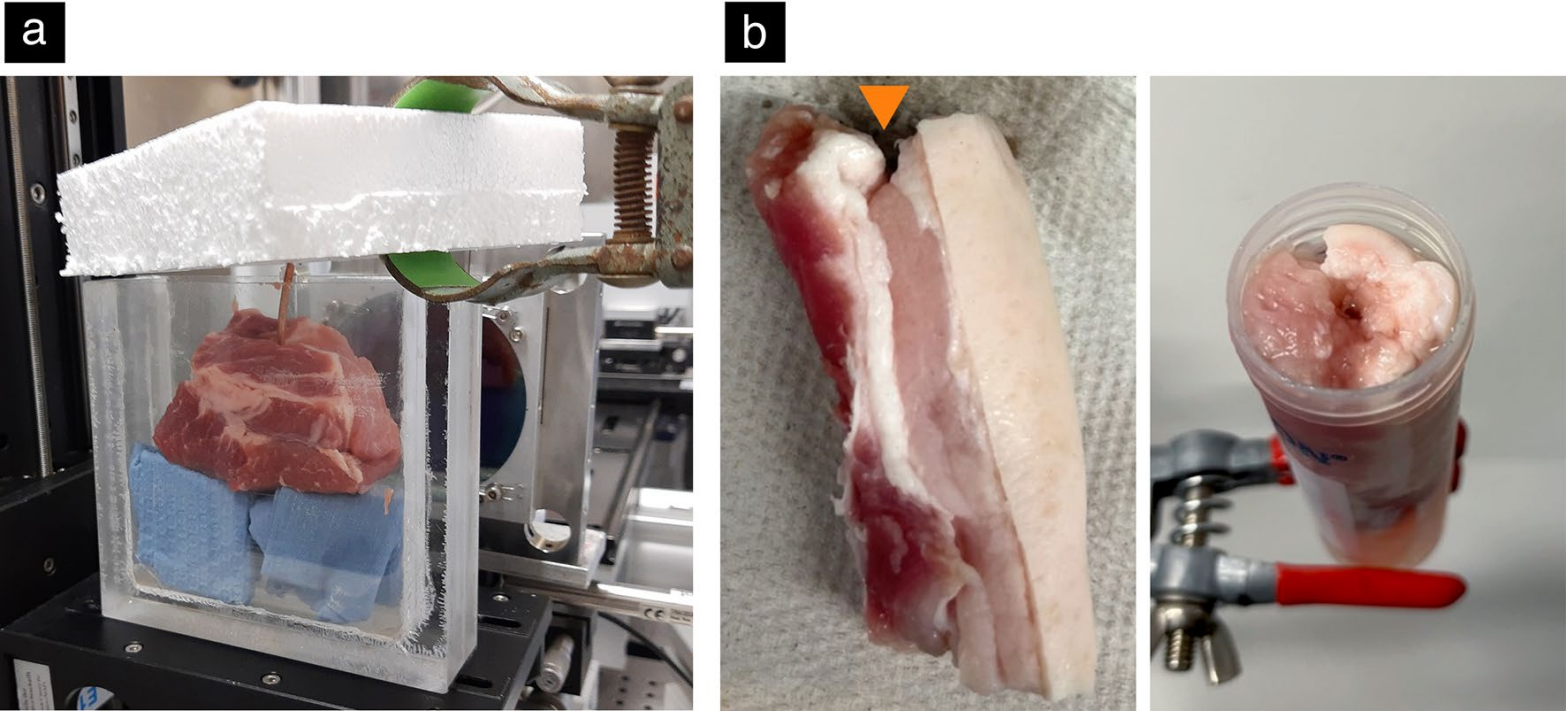
Photographs of the samples used in the experiments. (**a**) Piece of pork neck in measurement position, as used for the radiography experiment. The inserted copper needle is visible in the middle of the sample; it is cooled using the polystyrene basin on top of the polycarbonate sample holder. (**b**) Piece of pork belly used for the CT experiment after removal of the cooling tube. The orange arrow indicates the top-facing part of the sample.

For the radiographs, an unpreserved piece of pork neck approximately $8 \times 4 \times 3$ cm in dimensions is used. It is held in place using a holder made of two polycarbonate plates with an inner distance of 3 cm. The inserted copper needle has a diameter of 3 mm. The sample is shown in measurement position with the inserted needle in Fig. 8a.

A similar sample is chosen for the CT scans: A piece of unpreserved pork belly is cut to fit into a Falcon^TM^ tube (*Fisher Scientific GmbH*, Schwerte, Germany) 3 cm in diameter. It comprises comparably large sections of adipose tissue and muscle and additionally includes a layer of skin. The sample is shown in Fig. 8b.

### Dark-field calibration

In order to remove the spectrally induced dark-field signal caused by beam-hardening from the measurements, a calibration as described by Pelzer et al. is performed for the CT measurements^42^. For this purpose, different Polyoxymethylene (POM) blocks ranging in thickness from 7 to 75 mm are placed in the setup, replacing the sample tube in Fig. 1. POM is chosen to mimic the composition of the sample and is comparable to a 1:1-mixture of muscle and fat^43^. Radiographs with an exposure time of 4 s are acquired for each thickness, yielding a transmission and dark-field value. The measured dark-field values *D* and transmission values *T* in each the image are meaned and fitted using the empirical relation

2 $$\begin{aligned} D = a \cdot T^b, \end{aligned}$$

to obtain a look-up table. *a* and *b* are the fitting coefficients, for which the values 1.1 and 0.33 are obtained respectively when averaging over the used detector module for more reliable fitting. These values are used to correct the dark-field data in the CT scan using^43^

3 $$\begin{aligned} D^\text {(SAS)} \approx \frac{D}{D^\text {(sp)}(T, \varphi )}, \end{aligned}$$

where $D^\text {(SAS)}$ is the component of the dark-field signal representing small-angle scattering and *D* is the measured dark-field signal, $D^\text {(sp)}$ is the dark-field due to spectral effects, as determined in the calibration experiment. Due to the homogeneity of POM at micrometer length scales, it does not cause strong small-angle scattering—for this reason, it is assumed that all recorded signal stems from beam-hardening effects^43^. The dependence on the phase of the signal $\varphi$ is neglected due to the low visibility of $< 25\%$ of the interferometer^43^.

### Dose calculation

Because the porcine sample is similar in composition to a human breast, the dose in this work is given as the mean glandular dose (MGD). The MGD is calculated using the monoenergetic normalized glandular dose coefficients DgN(*E*) (with *E* the X-ray energy) as tabulated by Boone et al. for different compressed breast thicknesses^44^. Taking into account the spectrum of the rotating anode and summing over all energy bins *E*, this results in the following formula for the MGD^45^:

4 $$ {\text{MGD}} = \sum\limits_{E} K (E)[{\text{mGy}}] \cdot 0.114\left[ {\frac{{\text{R}}}{{{\text{mGy}}}}} \right] \cdot \left( {{\text{DgN}}({\text{E}})\left[ {\frac{{\text{R}}}{{{\text{mGy}}}}} \right]} \right). $$

Here, *K*(*E*) is the air kerma, the total sum of kinetic energy of all charged particles per unit mass in the first step of interaction. The air kerma is measured separately for the radiograph and CT setup by placing the clinical dosimeter (Diados T60005 MAM, *PTW Freiburg GmbH*, Freiburg, Germany) at the sample position and leaving all gratings in the beam path. Since the holder used in the radiography experiment contains a polycarbonate plate of 5 mm in thickness in front of the sample, such a plate is also placed in front of the dosimeter. The comparatively thin walls of the Falcon^TM^ tube are deemed negligible for the dose calculation; as such, no object is placed in front of the dosimeter for the air kerma measurement in the CT setup. The resulting air kerma values are ${0.166}\,\upmu \hbox {Gys}^{-1}$ and ${0.566}\,\upmu \hbox {Gys}^{-1}$ for the radiograph and CT setup respectively. A breast glandularity of 50% is assumed for all dose calculations since approximately half of each sample consists of fat.

## Data Availability

The datasets generated during and/or analyzed during the current study are available from the corresponding author on reasonable request.
